# Swedish Farmers’ Opinions about Biosecurity and Their Intention to Make Professionals Use Clean Protective Clothing When Entering the Stable

**DOI:** 10.3389/fvets.2016.00046

**Published:** 2016-06-22

**Authors:** Maria Nöremark, Susanna Sternberg Lewerin, Linda Ernholm, Jenny Frössling

**Affiliations:** ^1^Department of Disease Control and Epidemiology, National Veterinary Institute, Uppsala, Sweden; ^2^Department of Biomedical Sciences and Veterinary Public Health, Swedish University of Agricultural Sciences (SLU), Uppsala, Sweden; ^3^Department of Animal Environment and Health, Swedish University of Agricultural Sciences (SLU), Skara, Sweden

**Keywords:** theory of planned behavior, livestock, biosecurity, disease prevention, focus group

## Abstract

The study was part of a series of studies aiming to increase knowledge about spread and prevention of livestock diseases in Sweden. A specific biosecurity behavior, i.e., making professionals (e.g., veterinarian, repairman, livestock transporter) wear clean protective clothing when entering the stables was investigated through focus groups and a questionnaire survey. This behavior was seen as a proxy for other biosecurity behaviors. As part of questionnaire development, three focus group discussions with a total of 11 participating livestock farmers were held. The questionnaire was based on the model of Theory of Planned Behavior. Response was received from 2,081 farmers. In the focus groups, farmers expressed a willingness to provide visitors with clean protective clothing. However, some had experienced difficulties in making veterinarians use protective clothing, and mentioned a reluctance to correct their veterinarians. The participants mostly focused on diseases regulated by control programs, especially *Salmonella*. In parts, participants were well informed but some showed a lack of knowledge concerning routes of disease spread. They also mentioned external factors that made them deviate from biosecurity recommendations. Farmers called for biosecurity advice with focus on cost–benefit return. Among survey respondents, the intention to make visitors wear protective clothing was moderate. Analysis of underlying elements showed that a majority of farmers (88%) had a neutral attitude, i.e., they were neither in favor nor against this behavior. Measures of subjective norm indicated a varying degree of social pressure among respondents. However, the majority (63%) indicated a strong behavioral control, thus suggesting that they could make visitors use protective clothing if they wanted to. Although most farmers (84%) indicated a strong willingness to comply with the opinion of their veterinarians in biosecurity matters, 30% replied that their farm veterinarian is indifferent or negative toward making visitors use protective clothing. Demographic factors were significantly associated with the intention, and farmers with pigs, larger herds, and female farmers had a stronger intention. Regional differences were also found. The findings provide new insights into why farmers apply, or do not apply, biosecurity routines, and will be useful in the on-going work to improve farm biosecurity in Sweden.

## Introduction

Contagious livestock diseases can have a large negative impact on animal health, animal welfare, food production and, when it comes to zoonotic diseases, public health. One way to prevent introduction of disease is to apply effective biosecurity measures, such as avoiding direct animal contact between herds or using cleaning and disinfection to minimize the risk of indirect contact via fomites (1). The benefits of biosecurity were described long before the roles of bacteria and viruses were identified (2) and biosecurity measures can be used to prevent spread of both endemic and exotic diseases. Although this is old knowledge, it is not always implemented on farm level (3–7). The reasons for this gap between available knowledge and behavior are not fully known, but have received increasing attention within the field of veterinary research (8–18).

During the last 10 years, a number of studies have been performed in Sweden to investigate aspects relevant to the spread of disease between livestock farms; e.g., livestock trade patterns (19, 20), biosecurity routines (3), information uptake (21), frequency and spatial patterns of farm visits (22, 23), and biosecurity among professionals visiting farms (4). These studies have been focused on what is done, and not the reasons why. The current study is a continuation of the previous work, trying to identify factors influencing farmers’ biosecurity behavior and thereby increase the understanding of why there is a gap between available knowledge and biosecurity behavior.

Presenting research results to the industry and authorities can contribute to policy changes. One example is our studies of livestock trade (19, 20, 24), where the results contributed to the industry implementing a new health certification program for dairy cattle trade. There are several examples of situations when a better understanding of biosecurity behavior could be useful in future biosecurity work. In discussions with veterinary practitioners, the authors have encountered presumptions regarding farmers’ opinions and intentions considering biosecurity behavior. One example of this is the belief that investing in protective clothing for professionals visiting the farm would be too expensive for the farmer. A frequent strategy used by the Swedish veterinary authorities to improve farm biosecurity has been to send brochures and information letters to farmers (21). This is based on the belief (or hope) that providing information will increase knowledge and lead to altered behavior. However, human behavior is complex and according to behavioral theory, knowledge is not the only factor affecting actual behavior (25, 26).

**Figure 1:**
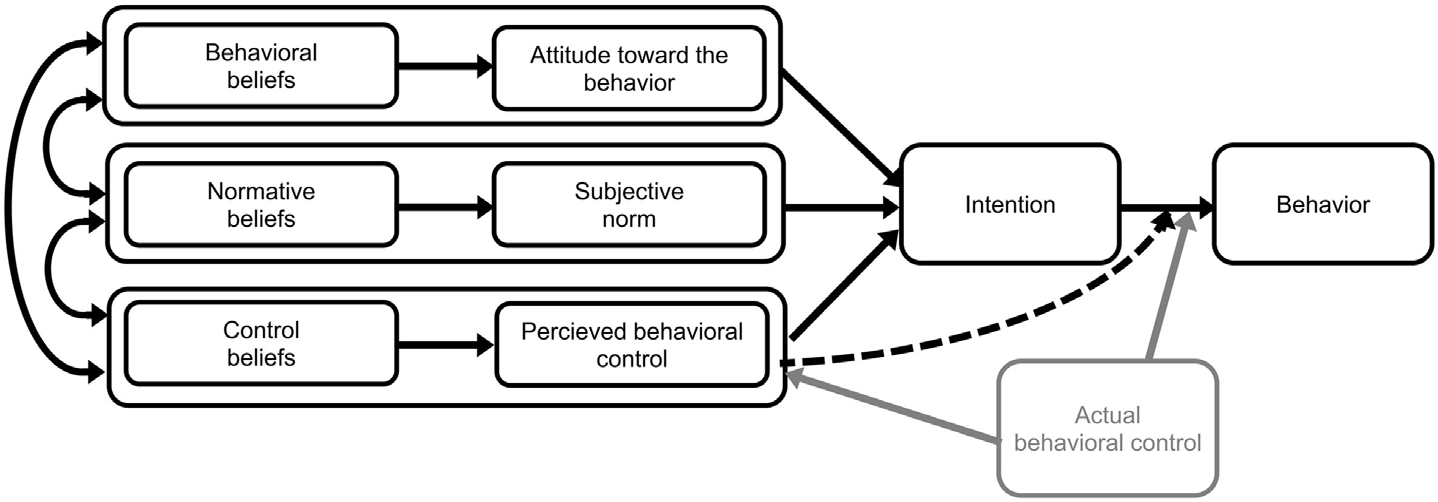
**TPB diagram, Copyright © Icek Ajzen 2006**.

Within the field of behavioral science, several different methods are used to investigate factors believed to determine final behavior, and these have been used to a limited extent within veterinary medicine. One model that had been used in some studies before this one (14, 27, 28) is the theory of planned behavior (TPB) developed by Ajzen (29). The theory behind the TPB-model is that behavior is a result of the intention to do something. In turn, this intention is affected by motivational factors: attitude (i.e., a positive or negative evaluation of performing the behavior), subjective norms (i.e., perceived social pressure to perform or not perform the behavior), and perceived behavioral control (i.e., perceived ease or difficulty to perform the behavior), see Figure 1 (30). The approach can be used to investigate one specific behavior.

The aim of this study was to investigate Swedish livestock farmers’ intentions toward the behavior to make professionals visiting the farm use clean protective clothing when entering the stables. This behavior was chosen as a proxy for preventive biosecurity measures. The overall goal was to increase the understanding of farmer behavior, which can be useful in future efforts to improve biosecurity on Swedish livestock farms.

## Materials and Methods

Data for this study were gathered through a questionnaire containing two parts, where results from analyses of the first part have been previously presented by Frössling and Nöremark (31). To gather qualitative data, and as part of questionnaire development, focus group discussions were held with farmers. The study involved Swedish livestock farmers and focused on farmers with cattle, pigs, sheep, goats, and to some extent poultry. The numbers and geographical distribution of livestock farms is reported annually by the Swedish Board of Agriculture and Statistics Sweden (32).

### Focus Group Discussions

#### Recruitment and Structure

Potential participants for the focus group discussions were identified with help from regional representatives of the Federation of Swedish Farmers who were asked to suggest farmers of different age, gender, and with different types of livestock and production. The authors then made a selection, striving for as demographically heterogeneous groups as possible, and contacted farmers by phone. When invited, the farmers were informed about the objectives of the study and they were also informed that their identity would not be revealed outside the group. Farmers who were willing to participate were sent written information about the study, again informing that the results would be treated anonymously. At the start of each meeting, a mutual agreement was made with the participants that they would treat what had been discussed in the room anonymously. The only compensation offered was reimbursement of travel costs. Three focus group meetings were held between May and August 2011, in Kalmar, Skara, and Uppsala. The total number of participants was 11. The initial plan was to have five meetings, also representing the most southern part and northern parts of Sweden, but only three of the regional representatives were able to suggest participants within the given time frame. Participants (listed in Table 1) were full-time farmers with medium-to-large herds (by Swedish standards) with dairy cattle, beef cattle, sheep, pigs, or poultry. Three farmers were female and eight were male. Their age ranged between ~30 and 70 years. Neither exact herd size nor age of each farmer was documented.

**Table 1:** **Participants of focus group discussions**.

Participant identification	Production type	Gender	Region
1	Sheep (educational setting)	Female	Skara
2	Beef cattle	Male	Skara
3	Dairy cattle	Male	Skara
4	Dairy cattle	Male	Skara
5	Pigs	Female	Kalmar
6	Poultry (broiler)	Female	Kalmar
7	Dairy cattle	Male	Kalmar
8	Beef cattle, pigs	Male	Kalmar
9	Dairy cattle	Male	Uppsala
10	Poultry (egg)	Male	Uppsala
11	Dairy cattle, pigs, poultry (broiler)	Male	Uppsala

The discussions were held in a semi-structured way (33, 34) and the duration was limited to 1 h 40 min. The reason for the time limit was that the authors deemed it easier to recruit participants if they were informed that the meeting would not take more than 2 h in total. After welcoming the participants, the purpose of the study and the format of the discussion were described. As an introduction, the farmers were asked to briefly present themselves and to share experiences from disease outbreaks. They were then encouraged to discuss as freely as possible, and along the way the study topics were introduced by the discussion facilitator (last author). The topics were as follows: protective clothing for farm visitors, purchase of livestock, construction and renovation of farm buildings, who you listen to when it comes to biosecurity, and a future biosecurity program. The reason that more topics than protective clothing were discussed was that the interviews were used also for developing other parts of the questionnaire. The last topic, a future biosecurity program, was supposed to be included in a questionnaire in collaboration with the animal health organizations, but the project was later canceled. The discussions were audio-recorded and a second researcher (first author) was also present to observe and take notes, in case the audio recording equipment would fail. All participants, including the researchers, had Swedish as their first language and the discussions were held in Swedish. Both researchers present have previously worked as field veterinarians, i.e., in close daily contact with farmers.

#### Compilation of Discussion Content

The recordings from the focus group discussions were transcribed. The transcripts and recordings were analyzed thematically (33, 34) by one of the authors (not present during discussions). The analysis was done manually and focused on identifying themes relevant for the questionnaire, and a full thematic analysis was not performed. A second author (present during the discussions) scrutinized the transcripts and themes, and further identified subthemes (33, 34). The identified themes were discussed in depth and confirmed with the other researcher present during the focus group discussions. Only themes raised and discussed by more than one participant were considered for the results presented in this paper. Apart from counting the number of times specific diseases were mentioned, no further quantification was done. The results shown in this article are not limited to the parts where protective clothing was specifically discussed, but deemed by the authors as relevant in relation to this type of preventive biosecurity behavior. Quotes that were considered representative of the views expressed were identified by two of the authors and the quotes were translated from Swedish by one of the authors. Translations were checked by all the other authors who compared the translations with the original text in the transcripts, and whenever in doubt also listened to the recordings.

### Questionnaire Study

#### Questionnaire Design and Structure

Prior to the focus group discussions, the researchers had preliminary ideas about areas and questions to include in the questionnaire study. The information obtained from the focus group meetings was used as an additional source of information and important for the refinement of the response alternatives in the questionnaire. The questionnaire design and structure is described in detail in Frössling and Nöremark (31), where the results of the first part of the questionnaire are also described. The second part of the questionnaire (52 questions) formed the basis of the study presented here. These questions dealt with the behavior to make farm visitors use clean protective clothing when entering the stable (make visitor use protective clothing). An English translation of the questionnaire, originally in Swedish, is available as an electronic supplement to this article.

The questions were constructed to roughly follow the TPB concept and to enable analysis of not only intention in itself but also of underlying components, such as attitude, subjective norm, and perceived behavioral control (35). This type of analysis requires that the behavior of interest is clearly defined, and that questions about obstacles, motivators, and other underlying factors are strictly related to the specific behavior. The behavior to make professionals visiting the farm use clean protective clothing when entering the stable (make visitors use protective clothing) was chosen as the behavior of interest because it had not been investigated before, and is of relevance for farmers with different livestock species or production. The behavior should not be seen as the most important biosecurity measure, but it can be seen as a proxy for other biosecurity behaviors. Intention was investigated through intention simulation, where farmers were asked how they would behave given eight different scenarios, i.e., indicate whether he or she would make visitors wear clean protective clothing when entering the stables or not. The remaining questions were designed to enable calculation of direct and indirect measurements of attitude, subjective norm, and perceived behavioral control (35), see Section “Statistical analysis of TPB components.”

#### Questionnaire Administration and Data Management

Selection of farmers, questionnaire administration, and data management were performed as previously described in Frössling and Nöremark (31). In brief, a dataset of all registered Swedish livestock holdings was retrieved from a national database at the Swedish Board of Agriculture. Farmers were selected by random sampling within each category of livestock species and the sample sizes for the respective strata were as follows: 1,800 cattle farmers, 800 sheep or goat farmers, 600 pig farmers, and 800 farmers with more than one species. Farmers with poultry were not included due to already known differences in production system and biosecurity level compared to other species. These sample sizes were roughly based on the total number of Swedish farms present within each category, the likelihood to get enough responses from each group of farmers, and the financial restraint to include a maximum of 4,000 farmers. The questionnaires were administered twice and were sent by mail in December 2012 and January 2013. Farmers were offered to respond by mail or online.

Questionnaire data were entered by single entry. Extreme values were checked and erroneous data entries were corrected. Responses regarding information about the farm, e.g., species present, production type, farm size, and future of the farm, were categorized. The questionnaire was designed and administered online using the web survey software Easyreseach (QuestBack International HQ, Oslo, Norway) and data were managed and analyzed using Stata (StataCorp. 2009. Stata Statistical Software: Release 13.0. College Station, TX: StataCorp LP.).

### Statistical Analysis of TPB Components

The direct measure of attitude was based on the mean score of responses to three questions (items). Internal consistency among items was tested by calculation of Cronbach’s alpha. Direct measures of subjective norm and perceived behavioral control were based on the mean score of responses to three and four questions, respectively.

The indirect measure of attitude (A) was calculated based on four items of behavioral beliefs and five outcome evaluations relating to these beliefs. In detail, it was calculated by multiplying the paired scores from each behavioral belief (a, b, c, d) and its corresponding outcome belief (e, f, g, h), and then calculating the sum of these products.

A=(a×e)+(b×f)+(c×g)+(d×h)

A positive result was interpreted as the respondent being in favor of the behavior (i.e., to make professionals visiting the farm use protective clothing), while a negative result was interpreted as the respondent being against this behavior. The indirect measure of subjective norm was based on six normative beliefs and six items of motivation to comply, relating to each source of social pressure. The indirect measure of perceived behavioral control was based on six control beliefs and six items of control belief power, relating to each belief. These indirect measures were calculated as described for the indirect measure of attitude. For subjective norm, a positive result was interpreted as the respondent perceiving a social pressure to perform the behavior, while a negative result was interpreted as respondent perceiving a social pressure not to perform the behavior. For perceived behavioral control, a positive result was interpreted as the respondent feeling in control of performing the behavior, while a negative result was interpreted as the respondent not feeling in control.

### Regression of Intention and Demographic Factors

Differences in proportions between different demographic groups were tested using a chi-squared test. Intention was based on the replies to the three scenarios simulating the intention to make a professional (livestock transporter, repairman, veterinary practitioner) visiting the farm uses clean protective clothing when entering the stables. The association between the intention and different demographic factors, or herd factors, was investigated using two-level ordered regression. In other words, the outcome variable was the intention indicated by the respondent, with values on the scale from 1 (no intention) to 7 (strong intention). Respondent was included as a random variable, using the xtlogit command in Stata, to account for replies to intention questions being repeated observations within respondent. Covariates were first tested using univariable regression and variables of potential interest (*P*-value < 0.25) were included as explanatory variables in a full multivariable model. Variables were then manually removed and reintroduced in a stepwise process until all remaining variables showed a significant association (*P*-value < 0.05) to the outcome. The variables tested as explanatory variables were as follows: farm type, region, number of full-time workers, future plan of production, production purpose, age, gender, and education level. The linear association between continuous variables and the outcome was assessed and such variables were included either as continuous, in categorized form based on percentiles, or as fractional polynomials, depending on which form had the best fit. The fit of alternative models was compared by calculation of the Akaike’s information criterion and the Bayesian information criterion. Models were not accepted as final if collinearity between variables or violation of the proportional odds assumption was indicated. In Stata, perfect collinearity between variables in a model is automatically tested and adjusted for as part of the regression command. However, collinearity diagnostic measures, such as the variance inflation factor, were also investigated. The proportional odds assumption of the models was tested using Brant’s test. For some models, outcome categories were collapsed into fewer categories in order to meet this assumption.

## Results

### Focus Groups

#### Protective Clothing, Benefits and Experienced Difficulties

Participants mentioned the benefit of protecting their own herd and a motivation to prevent spread of diseases to other herds. For citation reference, see Table 1.

Well they [visitors] think that I only want to protect my farm. But maybe I also want to protect the other ones, the farms they are going to next. (3)

The participants repeatedly emphasized that biosecurity measures that lead to an extra cost must give economic return. But none of the participants mentioned that the cost of buying protective clothing would be an obstacle, on the contrary the cost was stated to be minor.

It is no cost, a pair of boots and protective clothing costs nothing, not a damn thing. (7)

Participants with cattle pointed out that it is more difficult to make visitors wear protective clothing on cattle farms because stables are more “open,” often lacking a clear main entrance where a hygiene barrier can be set up. Although participants with pig and poultry reported that they in general did not have problems making visitors use protective clothing, there were also statements about the opposite.

When we let out the sows that were to be sent to slaughter, he was early and I wasn’t there when he arrived, and when I came out they [the sows] were gone. He just went in to fetch them, innovative but not clever, without changing his clothes. Then I told him off. (5)

Repeated comments were made about artificial insemination technicians and veterinarians preferring to use their own protective clothing instead of clothing provided on the farm. These visitor categories were identified as potential spreaders of disease. Participants also discussed difficulties in correcting the veterinarian for not using the provided clothing.

They [veterinarians] are the most difficult to get to, they are always ‘disease free’ [ironically], so then you have to point it out. (11)

I have ten pairs of rubber boots, one pair in each size, standing like this in a row … it works for a while, but then they become lazy … I have stopped nagging them. (3)

Really, the most important person to put protective clothing on is the vet, because he has definitely been in other herds with sick animals. (4)

We are dependent of them [veterinarians], if possible one doesn’t want to end up in a dispute (8)

A clear distinction between professionals and non-professionals coming to the farm was made by the participants. They discussed that Swedish consumers have limited knowledge about farming but that many want to visit farms, and various “open farm” activities often attract many visitors.

It is a great balancing act, at least to me. On the one hand I want good biosecurity, but we are also selling our products. We cannot turn this into something really big, so that the consumers may think that ‘this is just a huge source of infections’. (4)

#### Awareness of Sub-Optimal Biosecurity Behavior and Reasons for This

In the discussions, a number of situations were described when farmers are aware of best biosecurity practice, but despite that knowledge act differently.

From a biosecurity point of view it probably isn’t such a good idea to participate in a lot of cattle shows, but it is so damn fun. (4)

When we drive [the loader] between [different stables], with the wheels through manure, then it doesn’t matter if I change boots. (8)

Although participants expressed an awareness of financial losses caused by infectious diseases, farm economy was also described as a driver for deviating from preventive biosecurity measures.

But then you only see … what is standing there empty, only costing money. And, well, how much doesn’t it cost to have sick animals? One doesn’t think about that [when discussing benefits of regularly keeping stables empty for cleaning and disinfection]. (3)

Due to bank loans for investments in the farm, the banks were also repeatedly reported to have a strong influence on decisions that may affect farm biosecurity negatively.

You have a stable built for 100 cows and for some reason you only have 70 cows, and the bank stresses you to make sure the production goes up. I mean, then you are forced to disregard such things [biosecurity advice related to livestock trade]. (11)

#### Lack of Knowledge about Disease Spread

A general lack of knowledge regarding routes of disease transmission and their relative importance for different diseases was noticed.

But I don’t know at all how it spreads. One gets cautious just hearing the word ‘salmonella’. (3)

The virus thing. One can’t protect oneself against virus because it sort of exists everywhere. But, salmonella… If you don’t have common grazing, how could they possibly infect each other then? (6)

Well, virus diarrheas in dairy cattle… They enter in some way and, well, in the wintertime when my animals don’t meet any other animals… So of course the spread is airborne somehow. (7)

The opinion that it is good for the animals to be exposed to pathogens as this will make them immune was also mentioned in two different groups.

The more I protect myself, and the better I get, the more susceptible my animals become to different diseases. If I made my system totally closed… In some way it may be pretty good that my herd is in contact with seven other herds through neighboring pastures. It may be a way of keeping my animals healthy. (7)

#### Focus on Diseases in Specific Control Programs, Especially *Salmonella*

A main finding was that participants connect biosecurity and disease control to disease-specific programs, e.g., BVDV, but in particular the Swedish *Salmonella* control program. Large parts of the discussion in all groups were dominated by reflections and opinions related to *Salmonella* control. In total, the participants mentioned the word *Salmonella* 125 times (Skara 61, Kalmar 48, Uppsala 16). Diseases that are not included in specific control programs were rarely mentioned.

Yes, but we kind of think that if we protect ourselves from salmonella, then we have protected ourselves from most other things as well. (6)

To be labelled salmonella infected… It takes ten minutes before the whole region knows you are infected, and to get rid of that label takes 15 years, 20 years. (4)

…and not to do what is done now, throwing away five millions [SEK] on me, loads of problems and hassle and say that it [salmonella eradication on farm level] is completed. And then in ten years my farm will have salmonella again, because that’s what’s going to happen. This kind of waste of money really bothers me. (7)

If we should keep these strict salmonella rules, and we have them because of public health reasons, not for the sake of the animals. Often you cannot even notice that they have salmonella. Then, I find it very strange if we should bear all the costs for this. Costs which we don’t cause, and which we have no benefits from. (11)

#### The Farmer Collective and Reluctance to Question Others

When participants in the focus groups discussed future biosecurity programs, they referred to the whole farming community, not only discussing their own situation. They also stated that they experience that there is a peer pressure among Swedish farmers to participate in disease control programs offered by animal health organizations.

What we must do, we must raise the [farms with the] lowest level [of biosecurity]. (6)

The aim must be that, in the end, everyone should be a little bit better compared to where we were when we started. (4)

Well there are enough rules already, the main things is to get more people [farmers] to reach that level, not to create new rules. (8)

During discussions about herd health status, participants both expressed situations when they shared information, but also expressed that questioning someone else’s herd health status can be sensitive. One example was discussions about the benefits of standardized written health certificates when trading animals. Reluctance to question veterinarians was also expressed (see above).

Most people are considerate of others. Well, there are exceptions, but most people ask, not in a mean way, but if your animals have come together [after breaking through the fence] the spontaneous question is ‘Are you free from BVD?’ (9)

So that I don’t have to stand there and question [someone]. [benefits of written health certificates] (4)

#### Role of Veterinarians

Some participants had experienced veterinarians who were not interested in biosecurity or preventive work, but several positive experiences were also reported. It was clear that the participants wanted the veterinarian to have an advisory role. They wanted financial incentives for implementing biosecurity measures, and emphasized the need for veterinarians to have an understanding of the financial reality of the farm.

Then there are different vets, some vets are not so interested in animal health prevention. But, and the same goes for doctors, I mean, they see as their mission to cure and not to prevent. (11)

They don’t have, well, the balance, the economy… They don’t understand it. It seems like that is something, economy is something they don’t learn in vet school. (9)

And then, maybe you will be able to … choose to always have the same person [veterinarian] coming to the farm, someone who gets to know both the person in charge, and the stables and the animals. To get sort of, a more uniform sounding board. (9)

That someone points out the problems, and then you discuss these together to find a solution. The solutions look different, they are unique for each farm. I see a risk if you only have your farm vet, that you become a bit blind to your own flaws. Well it happens to you and of course it happens to your advisors as well. So it can be good if someone comes and sees with fresh eyes, but it needs to be someone who has the ability to enthuse. (11)

### Questionnaire

#### Response

The total number of replies to the questionnaire was 2,081. The response rate among farmers that responded to the mailed questionnaire was 52% overall, and 26% for farmers with cattle, 30% for farmers with pigs, 61% for farmers with small ruminants, and 26% for farmers with mixed species. The questionnaire replies from respondents that reported at least one animal of the relevant species on the farm and responded to the TPB section of the questionnaire were included in a dataset for further analysis (*n* = 1,890). By each section, there was a gradual decline in the number of respondents that provided a reply to all questions within the section. The number of full responses was 1,727 for the intentions scenarios further investigated using ordered regression. The corresponding numbers for direct and indirect measures of intention components were 1,778 and 1,710 for attitude, 1,799 and 901 for subjective norm, and 1,763 and 628 for perceived behavioral control.

#### Description of Respondents

In total, the numbers of respondents from farms for each species were as follows: cattle farms, *n* = 1,010; pig farms, *n* = 180; sheep or goat farms, *n* = 481; and mixed species farms, *n* = 219. The reported numbers of animals on the farms are summarized by species and animal category in Table 2. The geographical distribution of replies corresponded roughly to the distribution of farms in the country, with more and relatively larger farms in the south. Cattle farmers were the most frequent respondents from all regions (~45–60%) and two-thirds of respondents with pig herds were located in South Sweden or West Sweden (Nomenclature of Territorial Units for Statistics (NUTS) level 2) (36). In 53% of the farms, there was less than one person working full time on the farm. The 95th percentile and maximum number of full-time workers were 3 and 14, respectively. According to the majority of respondents, their farm production would remain the same (47%) or increase (19%) in the next 5 years. Almost one-third (27%) believed that their production would decrease or stop within 5 years.

**Table 2:** **Number of herds with average number of animals by species and production type, in a questionnaire study investigating farmers’ intention to make professionals entering the stable use protective clothing (Sweden, 2012–2013)**.

Production type	Number of herds	Number of animals			Animal category
		Median	Percentile		
			25th	75th	
Cattle					
Dairy	432	60	32	100	Dairy cows
Beef, suckler	569	14	7	25	Cows
Beef, calves for slaughter	134	20	7	50	Slaughtered cattle per year
Other	87	7	3	25	Cattle
Total	1,222				
Pigs					
Breeding	10	188	120	220	Sows
Multiplying	47	110	40	288	Sows
Pool	21				
Nucleus		1,200	880	1,800	Sows
Satellite		4,500	2,500	5,500	Piglets per year
Integrated	60	185	70	300	Sows
Slaughter	76	2,550	370	4,750	Slaughtered pigs per year
Total	214				
Small ruminants					
Sheep	617	12	7	25	Ewes
Goats	62	4	2	9	Goats
Total	679				

The majority of respondents were owners of the farm (94%). Overall, the proportions of men and women among responders, indicating their gender, were 70 and 25%, respectively. However, the proportions differed by farm type and for sheep and goat farms almost half of the respondents (44%) were women. In addition, the education level of respondents varied by gender. For example, 41% of female respondents and 21% of male respondents had a higher education (university or equivalent). In total, 42% had an education focused on agriculture. Most respondents (29%) were 51–60 years old and 75% were 41–70 years old. The majority of respondents (78%) had worked with their current species of animals for more than 10 years. According to 37% of respondents, the purpose of their livestock production, or employment at a livestock farm, was to make a living, while 24% responded that their livestock farming was pure hobby. The purpose of livestock farming differed between species, where the majority of cattle farmers (52%) and pig farmers (76%) had their production to make a living, while only 5% of sheep and goat farmers kept their animals for this purpose. Instead, 56% of sheep and goat farmers indicated that their animal production was pure hobby.

Protective clothing for visitors was provided on the farm according to 47% of the respondents. This proportion differed considerably among farm types (chi-squared test, *P* < 0.001); Cattle dairy 83%, Cattle other 32%, Pigs piglet 92%, Pigs fattening 88%, Small ruminants 21%, and Mixed 44%.

#### Intention and Behavioral Predictor Components

**Figure 2:**
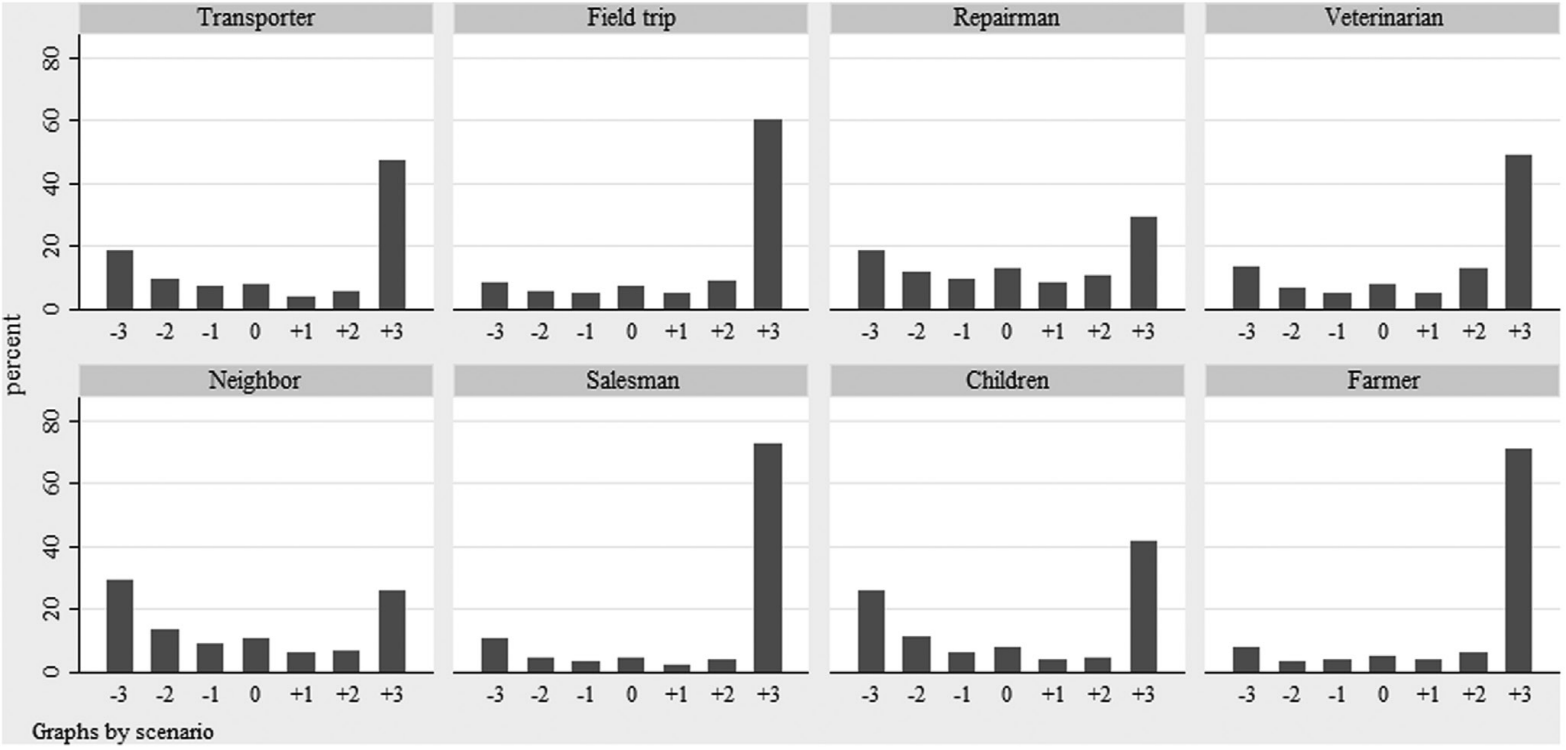
**Distribution of farmers’ intention to make different categories of farm visitors use clean protective clothing when entering the stables**. The intention was indicated on a seven-grade scale ranging from low intention (−3) to strong intention (+3). The survey was based on a questionnaire and included farmers with cattle, pigs, or small ruminants, from all parts of Sweden (2012/2013).

**Figure 3:**
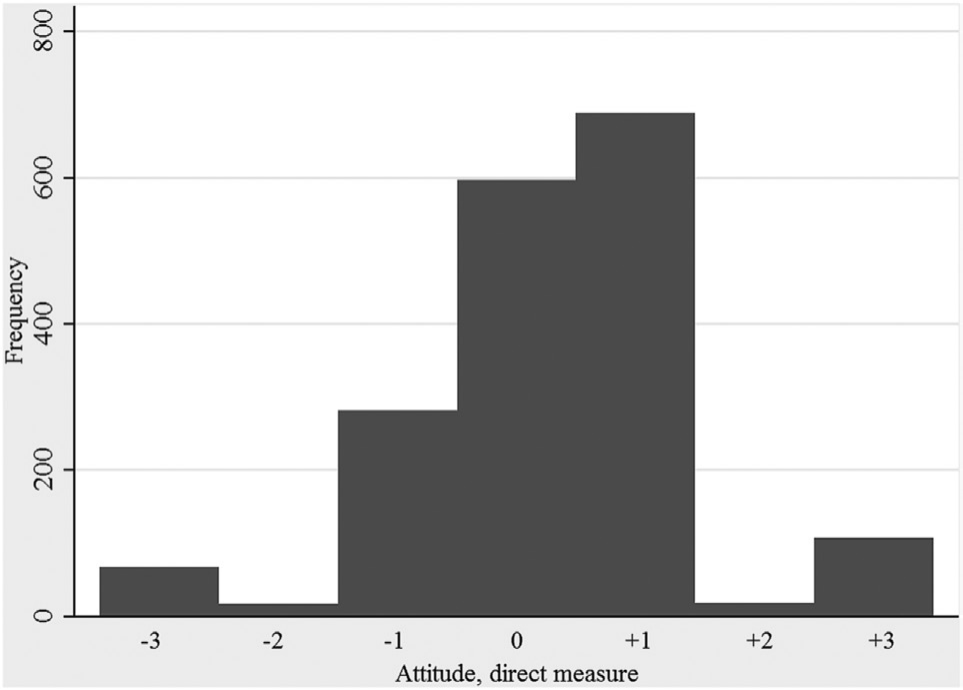
**Distribution of direct measures of attitude toward making professionals visiting the farm use clean protective clothing when entering the stables**. A positive result is interpreted as the respondent being in favor of the behavior, while a negative result is interpreted as the respondent being against this behavior. The results are based on responses to a questionnaire survey, including farmers with cattle, pigs, or small ruminants, from all parts of Sweden (2012/2013).

**Figure 4:**
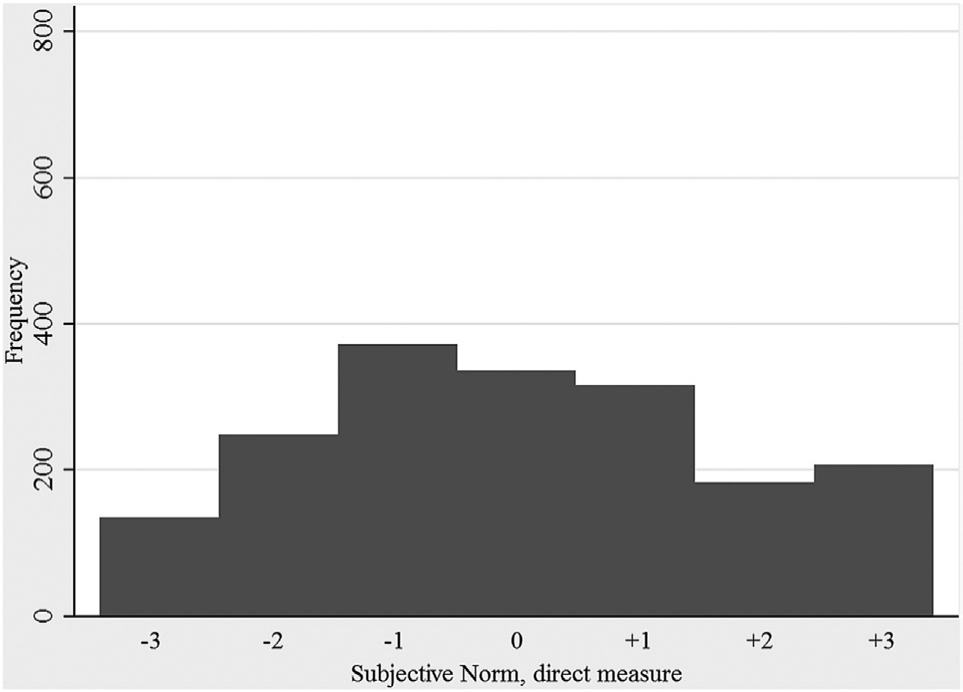
**Distribution of direct measures of subjective norms related to making professionals visiting the farm use clean protective clothing when entering the stables**. A positive result is interpreted as the respondent perceiving a social pressure to perform the behavior, while a negative result is interpreted as the respondent perceiving a social pressure not to perform the behavior. The results are based on responses to a questionnaire survey, including farmers with cattle, pigs, or small ruminants, from all parts of Sweden (2012/2013).

**Figure 5:**
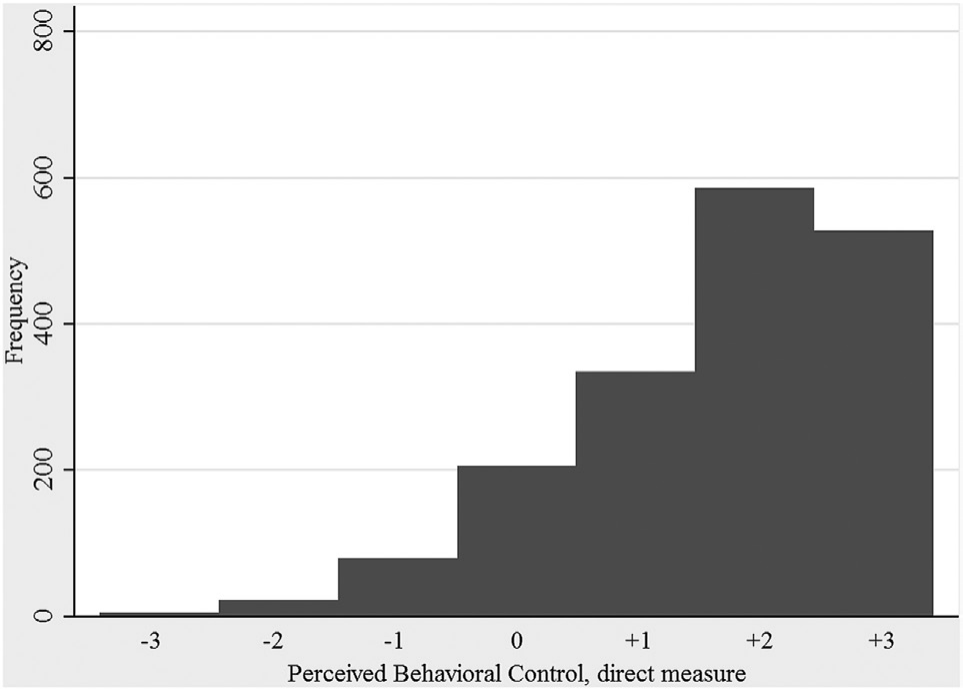
**Distribution of direct measures of perceived behavioral control to make professionals visiting the farm use clean protective clothing when entering the stables**. A positive result is interpreted as the respondent feeling in control of performing the behavior, while a negative result is interpreted as the respondent not feeling in control of performing the behavior. The results are based on responses to questionnaire survey, including farmers with cattle, pigs, or small ruminants, from all parts of Sweden (2012/2013).

The distribution of farmers’ intention to make different categories of farm visitors use clean protective clothing is shown in Figure 2. The median of respondents’ average intention to make visitors wear protective clothing was 5.3 on a 7 grade scale (5th and 95th percentiles: 1.9 and 7). However, the indicated intention differed depending on the scenario given and was highest for the scenarios that involved a salesman without protective clothing and an acquaintance with a herd of the same species as the farmer, who paid a visit during a period when infectious disease seemed to be circulating (average intention; 6.5). The lowest average intention (3.7) was seen for the scenario where the visitor was a new neighbor who just started small-scale production with the same species as the farmer. The distributions of direct measures of attitude, subjective norm, and perceived behavioral control are shown in Figures 3–5. The median measure of attitude was 0, and most farmers (88%) indicated a relatively neutral attitude (−1 to +1) toward making professional visitors wear protective clothing, i.e., they were neither in favor nor against this behavior. The median direct measure of subjective norm (0) indicated a lack of social pressure to perform, or not to perform, the behavior. However, this measure was evenly distributed across the scale, with 8–21% of respondents in each response category. A majority of farmers (63%) indicated a strong perceived behavioral control (≥ +2) to perform the behavior (median: +2).

The internal consistency for items used to calculate the direct measures of attitude, subjective norm, and perceived behavioral control, was 0.69, 0.75, and 0.68, respectively. The correlation between direct and indirect measures was 0.29 for attitude, 0.70 for subjective norm, and 0.44 for perceived behavioral control. Farm veterinarian was the category whose normative beliefs the respondents indicated the strongest motivation to comply with (≥ +2: 84%). However, 31% of respondents indicated that they perceive their farm veterinarian as indifferent or negative toward the farmer making visitors use protective clothing. Farmers’ mean response for motivation to comply was 6.3 for their farm veterinarians, 5.9 for their employers (if employed), 5.5 for industry policies, 4.9 for close friends and family, and 4.7 for neighbors (all on a 7-grade scale).

#### Regression Results

Results from univariable and multivariable regression are presented in Table 3. The variables that were significantly associated with intention in the final multivariable model were farm type, region, number of full-time workers, age, and gender.

**Table 3:** **Output from univariable and multivariable ordered logistic regression models used to investigate the intention to make a professional (veterinarian, repairman, or transporter) visiting the farm wears clean protective clothing and potential associations with demographic factors and herd factors**.

Explanatory variable category	Univariable regression				Multivariable regression			
	OR	95% confidence interval		*p*-value	OR	95% confidence interval		*p*-value
Farm type								
Cattle, dairy	*Reference category*			<0.001	*Reference category*			<0.001
Cattle, other	0.74	0.56	0.98		1.03	0.76	1.41	
Pigs, piglets	15.81	9.31	26.85		13.93	8.25	23.54	
Pigs, fattening	3.28	1.75	6.14		4.10	2.19	7.67	
Small ruminants	1.09	0.81	1.48		1.38	0.98	1.94	
Mixed	1.06	0.73	1.52		1.27	0.88	1.85	
Region^a^								
South	*Reference category*			<0.001	*Reference category*			<0.001
South East	0.27	0.18	0.38		0.38	0.26	0.53	
West	0.60	0.43	0.85		0.68	0.49	0.95	
East Middle	0.61	0.42	0.87		0.68	0.48	0.96	
North	0.65	0.45	0.94		0.85	0.60	1.21	
Number of full-time workers	1.25	1.15	1.36	<0.001	1.19	1.08	1.30	<0.001
Future plan of production								
Sustain or increase production	*Reference category*			0.889				
Cease production	0.98	0.76	1.26					
Production purpose^b^	1.08	1.04	1.13	<0.001				
Age^c^	0.50	0.30	0.83	0.007	0.41	0.26	0.67	<0.001
Gender								
Female	*Reference category*			<0.001	*Reference category*			<0.001
Male	0.58	0.45	0.74		0.59	0.46	0.76	
Education level								
Compulsory school (9 years)	*Reference category*			0.001				
Upper secondary school	0.98	0.75	1.30					
University or equivalent	1.58	1.15	2.17					

*Intention was based on respondents’ replies to three intention scenarios and the models were leveled with multiple responses (*n* = 5,134) from each respondent (*N* = 1,759, Sweden 2012–2013)*.

*^a^Categories were based on Nomenclature of Territorial Units for Statistics (NUTS) level 2*.

*^b^Continuous variable, ranging from “hobby” (lowest) to “make a living from production” (highest)*.

*^c^Continuous variable indicating age by 10-year category (x), transformed to x^−2^*.

## Discussion

Among the livestock farmers in this study, the intention to make visitors use protective clothing was moderate, and among some categories of farmers, low. Based on the results, the reason for this does not appear to be a perceived lack of behavioral control, but rather an absence of conviction that this behavior is of importance, i.e., attitude. In addition, the perceived pressure from others to perform the behavior of interest (subjective norm) varied and was low according to many farmers. In the focus group discussions, there were indications that farmers may be reluctant to ask, e.g., the veterinarian to wear protective clothing because they think that veterinarians are highly educated and should know what they are doing, or because they are dependent on them and do not want to risk a dispute. However, in the questionnaire, many farmers reported a strong perceived behavioral control. In other words, although they believe they can make visitors use protective clothing, it seems to be lack of belief that protective clothing will have a positive effect, which decreases their intention.

The findings of low-to-moderate intention seem to contradict results from the first part of the questionnaire, where a large majority of farmers responded that it is very important to protect their herd from infectious diseases (31). One explanation to why some farmers have a low intention to make visitors use protective clothing, even though they want to protect their herd against infections, may be that they do not see the benefits of protective clothing. It has been shown that insufficient knowledge about routes of disease transmission, which was also a finding from the focus group discussions, may play a part in this (37). If farmers believe, as observed during the focus group discussions, that a disease is caused by infection that is air-borne over larger distances, when in truth it is not, it is understandable that some farmers fail to see the benefits of clean protective clothing. Raising the general knowledge regarding disease spread will not by itself change behavior, but understanding disease spread may prevent some of the inconsistent biosecurity behavior previously observed (21). Another theory, based both on the focus group discussions and comments in the questionnaire, could be that farmers trust that the visitors themselves take responsibility for not spreading disease. This is a relevant finding seen in relation to a study of professionals, e.g., veterinarians and hoof trimmers where in contrast, it was shown that they expect the farmer to take responsibility for biosecurity (4).

The direct measure of subjective norm did not reveal a strong social pressure to make visitors use protective clothing, but also showed a large variation. Analysis of indirect measures of subjective norm indicated that many farmers care about what their farm veterinarian thinks about their biosecurity behavior. However, one-third of the farmers did not believe that their farm veterinarians think it is important to make visitors use protective clothing. This finding is supported by results from the focus group discussions and by questionnaire free-text comments with testimonies of insufficient biosecurity among veterinarians (results not shown). This is also in agreement with previous studies, where it was found that veterinary practitioners question the benefits of biosecurity (10, 38).

Multivariable analysis indicated that the intention to make visitors use protective clothing differs among different categories of farmers. Pig farmers had a higher intention to make visitors use protective clothing compared to farms with other species, which is consistent with previous studies where general biosecurity has been observed to be better in pig farms compared to farms with other species (21, 39, 40). The proportion of farmers who responded that protective clothing for visitors was available on the farm also differed between farmers with different species of animals in a similar way. To make protective clothing easily available for visitors can be seen as yet another reflection of the farmer’s intention to make the visitor use protective clothing. The finding that pig farmers have a relatively higher intention is probably highly influenced by the strict separation of batches usually practiced in modern pig farming. In addition, there may be a remaining effect of the eradication campaign for Aujeszky’s disease, where the importance of protective clothing for visitors was stressed and it was made mandatory for all pig farms to provide such clothing. Similar control programs in other species, e.g., the eradication campaign for bovine viral diarrhea in cattle and the control program for Maedi Visna in sheep, have not included requirements to provide protective clothing for visitors. The farmers in the focus groups did not consider providing protective clothing costly and the measures of perceived behavioral control were very high in this study. This indicates that putting a stronger demand on farmers to provide protective clothing also in control programs for cattle and sheep diseases could influence the attitude and behavior of these categories of farmers in a positive way. From the focus group discussions, there were also clear tendencies to put trust in specific programs or testing, and that measures taken within the programs were sufficient. The *Salmonella* control program in Sweden, which was the main topic for many farmers in the focus groups does not include requirements for protective clothing for visitors. The strong focus on *Salmonella* may be explained by the long tradition to control *Salmonella* in Sweden, and farmers’ fear of increased work load and costs caused by disease control intervention if *Salmonella* was detected in their animals.

In Sweden, there is a tradition to use collective efforts to control infectious diseases in the livestock populations. Examples of diseases that have been eradicated are Aujeszky’s disease in pigs, and in cattle; bovine brucellosis, bovine tuberculosis, infectious bovine rhinotracheitis, and bovine viral diarrhea (41). These programs have had a strong involvement by the farmers’ organizations and the industry. Many dairies and slaughterhouses have been cooperatively owned and these factors may explain why farmers in the focus groups repeatedly spoke not only about themselves but also about all farmers. Continuing this tradition of including the farmers’ organizations in future efforts to increase biosecurity is likely to be beneficial. However, although the focus group participants expressed positive peer pressure, collective thinking, and situations when they feel it is natural to discuss herd health status, the fear of openly questioning others was raised. Clarifying responsibility and involvement of all stakeholders; industry, authorities, and veterinarians could potentially help overcome such problems.

Analysis of intention detected some regional differences, with significantly higher intention to make visitors use protective clothing in the south part of Sweden. One possible explanation could be that the south of Sweden has a higher density of farms compared to the rest of the country. This may in turn contribute to a higher perceived risk of disease introduction and, as a consequence, a higher intention to prevent introduction. The intention was lower in the south east of Sweden and the reasons why are not known. However, there is a history of sharing pasture in this region and in that context some farmers question the relative contribution of making visitors use protective clothing to the overall biosecurity level of their farm (personal communication, Estelle Ågren). Furthermore, farmers in this region also reported lower perceived knowledge about disease spread (31).

The number of full-time workers on the farm was positively associated with the intention to make visitors use protective clothing. The number of employees is an indirect measure of farm size, which is also related to the number of professionals visiting the farm (23). A possible theory is that larger farms are more “professional” or that farms with several persons working on the farm need to have clear routines in place. Age was also significantly associated with the intention, where older respondents in general indicated a higher intention. In addition, older farmers are more adverse toward buying animals with an unknown health status (31). It is unknown whether this is the result of an age effect or a generation effect. In other words, it is possible that the difference among age categories indicates an on-going change in opinion and that the younger generation of farmers has a lower awareness of biosecurity. In previous studies, Swedish veterinarians have reported a perceived general decline in biosecurity (4), but this would need to be further investigated. Consistent with results from the first part of the questionnaire (31), female farmers reported a higher intention compared to male farmers. Correspondingly, significant differences between genders have been observed among Swedish pig farmers, where the female gender was associated with higher biosecurity (42). Similar findings have also been reported in studies related to human health (43, 44).

The overall response rate in the questionnaire part of this study was comparable to, or higher than, previous studies on similar topics in Sweden (3, 28). However, the respondents’ willingness to reply seems to have been decreasing toward the end of the questionnaire. The TPB design requires some repetition and similarity among questions and this was explained at the beginning of the questionnaire. Nevertheless, it was clear from the free-text comments that some farmers experienced the last TPB part of the questionnaire repetitive and tedious to fill in. It cannot be excluded that farmers interested in biosecurity were more likely to respond to the questionnaire, or that this response bias was stronger for the last part of the questionnaire. The TPB method has been extensively used, but the validity of the method has also been criticized (45). However, despite limitations of the method, and the fact that some responses were incomplete, we find the information from this investigation useful. Separating the effect of attitude, subjective norm and perceived behavioral control clearly indicated that the main reason behind farmers’ low intention to perform strict biosecurity behavior is the attitude, i.e., they do not have a favorable evaluation of the behavior. This finding is useful in on-going work to improve farm biosecurity in Sweden.

In the focus group discussions, the groups are not meant to be representative but rather just as divergent as possible, so that as many views and opinions as possible are captured. This study only included three focus groups with a total of 11 participants and there may be important opinions that we failed to identify. A clear limitation was that only full-time farmers with medium-to-large herds were recruited for the study, and no hobby farmers. However, many opinions were repeated several times and this was consistent between focus groups. Due to the non-representative selection of participants, and the small number of participants, comparisons or quantification of differences between categories of farmers could not be made.

As mentioned above, minimizing direct animal contact and indirect contact via fomites, such as contaminated equipment or boots, are means of preventing spread of infectious diseases. From previous studies (3, 19, 23) and knowledge about the industry structure, it is known that there are major differences between farms with different species and production types, regarding, e.g., livestock trade and other aspects relevant for disease prevention. To enable comparison between different farm types, there was a need to identify a situation that is relevant for largely all types of farms. In this case, the behavior to make professionals visiting the farm use clean protective clothing when entering the stable was chosen. This should not be seen as the most important biosecurity behavior, but rather as a proxy for different biosecurity-related behaviors.

Although not investigated, we expect that deficiencies in other types of biosecurity behavior may also in part be due to lack of belief in the benefits. Motivation is one of several components that are expected to influence behavioral change (8, 25, 46). Based on the results from this study, and as previously concluded by Laanen et al. (40), it seems logical that farm veterinarians should be engaged in the work to motivate farmers to implement farm biosecurity by communicating the benefits of biosecurity behavior (attitude change) and by affecting the perceived subjective norm. However, previous studies have identified gaps between which biosecurity measures farmers and veterinary practitioners believe to be effective (47). Also, the practitioners themselves need to be motivated and trust that biosecurity is beneficial for farm health. A new national biosecurity program (www.smittsakra.se) has recently been launched in Sweden and this provides a framework for future focus on these issues. Continued work is needed to increase motivation and facilitate the implementation of biosecurity measures.

## Author Contributions

JF, MN, and SL designed the study. JF and MN recruited participants, conducted focus group discussions, and designed the questionnaire. JF, LE, and MN analyzed the data from the discussions and JF and MN analyzed the data from the questionnaire study. JF and MN drafted the manuscript, all authors critically revised the manuscript.

## Conflict of Interest Statement

The authors declare that the research was conducted in the absence of any commercial or financial relationships that could be construed as a potential conflict of interest.
