# Supplementary material for: Swedish Farmers’ Opinions about Biosecurity and Their Intention to Make Professionals Use Clean Protective Clothing When Entering the Stable
Source: Front Vet Sci. 2016 Jun 22;3:46. doi: 10.3389/fvets.2016.00046 (PMC4916181; doi:10.3389/fvets.2016.00046)
Supplement: Supplementary file 1 [file Presentation_1.pdf]

# Questionnaire about hygiene and disease control in livestock

**Information and instructions**

This questionnaire is aimed for farm owners and farm workers who handle cattle, pigs, sheep, goats or poultry. We are interested in responses from farms of different sizes.

The questionnaire takes about 15 minutes to answer and deals with perceptions, motivations and barriers to hygiene and infection control in Swedish herds. We are not searching for right or wrong answers, but we genuinely want to know how you handle things in practice and how you perceive different situations. The results will provide a basis for improving efforts to prevent and control infectious diseases in livestock.

The second half of the questionnaire deals with protective clothing and persons visiting farms as part of their profession. It is based on a behavioural science method, and many questions may seem similar. This is however how it is meant to be.

The questionnaire does not cover all the different routes of infection, such as trade with live animals, because this is examined in other studies.

In many of the questions you will be asked to give your response on a seven-point scale, e.g. from 'strongly agree' to 'strongly disagree'. Tick the option you spontaneously feel closest to your opinion or behaviour. If you do not have a clear view, select the middle option. If there is a question that you feel that you cannot answer, leave it blank and move on to the next.

Please note that your reply to this questionnaire is completely anonymous.

## A. The FARM and YOUR BACKGROUND

**Enter the number of animals of different production types on the farm. An approximate number is OK. If the stables are just temporarily empty (e.g. between batches), enter how many animals that are usually present.**

**1. Cattle, fill in the number of animals on the line that matches the farm production type**

| | | |
|---|---|---|
| Dairy production | ☐ | Number of dairy cows |
| Beef and/or suckler cows | ☐ | Number of cows |
| Purchase of calves for fattening | ☐ | Number of animals sent to slaughter each year |
| Other type of production | ☐ | Number of cattle |

**2. Pigs, fill in the number of animals on the line that matches the farm production type**
e.g. if the farm has pigs for fattening, just fill in the number of pigs slaughtered per year, but if the farm is integrated, fill in both the number of slaughtered fattening pigs and the type of piglet production.

| | | |
|---|---|---|
| Breeding or gilt production | ☐ | Number of sows |
| Multiplying | ☐ | Number of sows |
| Fattening | ☐ | Number of fattening pigs slaughtered each year |
| Sow pool centre | ☐ | Number of sows |
| Sow pool satellite | ☐ | Number of piglets produced each year |

**3. Sheep and goats**

| | | |
|---|---|---|
| Sheep | | Number of ewes |
| Goats | | Number of does |
| Lambs or kids | | Annual number |

**4. Poultry, fill in the number of animals on the line that matches the farm production type**

| | | |
|---|---|---|
| Breeding | | Number |
| Egg production | | Number of hen places |
| Broiler | | Number of slaughtered birds per year |
| Hobby | | Number |
| Other types of poultry | | Number of birds |

**5. Exotic ruminants (e.g. bison, wisent, camel, alpaca, llama)**

Fill in species and total number (i.e. count females, males and young animals)

______________________________________________________________________________

______________________________________________________________________________

______________________________________________________________________________

______________________________________________________________________________

**6. Is the farm's livestock production certified organic?**

- ○ No
- ○ Don’t know
- ○ Yes, fill in type of certificate system below

Certificate:

______________________________________________________________________________

**7. If you have comments to the questions above, please write here**

______________________________________________________________________________

______________________________________________________________________________

______________________________________________________________________________

______________________________________________________________________________

**8. In what county is the farm located?**

- Blekinge
- Dalarna
- Gotland
- Gävleborg
- Halland
- Jämtland
- Jönköping
- Kalmar
- Kronoberg
- Norrbotten
- Skåne
- Stockholm
- Södermanland
- Uppsala
- Värmland
- Västerbotten
- Västernorrland
- Västmanland
- Västra Götaland
- Örebro
- Östergötland

**9. What is you role on the farm?**

- Owner
- Employed foreman
- Employed
- Other

**10. How many people work with the animals on the farm, including yourself?**

Number of persons full time

Number of persons part time

**11. How old are you?**

- 30 or younger
- 31-40
- 41-50
- 51-60
- 61-70
- over 70

**12. What is your gender?**

- Woman
- Man
- Prefer not to fill in

**13. Enter your education level**

- Compulsory school
- Upper secondary school
- University or equivalent

**14. Were any of these educations programs agriculture oriented?**

- Yes
- No

**15. If you have comments to the questions above, please write here**

**16. How many years have you been working with the animal species present on the farm?**
If there are multiple species, specify the time for the species that you have work longest with

- ○ 0-5 years
- ○ 6-10 years
- ○ 10-20 years
- ○ More than 20 years

**17. If you own the farm, what is the purpose of the livestock production?**

Keeping livestock is a hobby ○ ○ ○ ○ ○ ○ ○ I keep livestock to make a living

**18. I you are the owner of the farm, what is your plan for the livestock production in 5 years from now?**
(specify one or more options)

- ☐ Production is discontinued
- ☐ I am about to discontinue production
- ☐ The number of animals has been decreased
- ☐ Production is the same as today
- ☐ I have increased the number of animals
- ☐ I have changed the type of production
- ☐ A successor has taken over

**19. If you are employed, do you plan to stay within this profession in 5 years from now?**

Yes, I want to continue ○ ○ ○ ○ ○ ○ ○ No, I want to quit

**20. If you have comments to the questions above, please write here**

______________________________________________________________________________
______________________________________________________________________________
______________________________________________________________________________
______________________________________________________________________________

## B. INFECTIOUS LIVESTOCK DISEASES

**21. To keep the herd free from infectious diseases is very important for me**

Totally disagree ○ ○ ○ ○ ○ ○ ○ Totally agree

**22. If the animals are regularly exposed to infections, they will become more resistant and have less disease**

Totally disagree ○ ○ ○ ○ ○ ○ ○ Totally agree

**23. If infectious disease would spread from my herd to other herds, there would be negative consequences for me**

Totally disagree ○ ○ ○ ○ ○ ○ ○ Totally agree

**24. An outbreak of infectious disease in my herd would have negative effects on my economy**

Totally disagree ○ ○ ○ ○ ○ ○ ○ Totally agree

**25. Has the farm experienced an outbreak of infectious disease that seriously affected the animals or production?**

Many times ○ ○ ○ ○ ○ ○ ○ Never

**26. Do you know how different infectious diseases spread and what you can do to prevent introduction of infections into the herd?**

Do not know at all ○ ○ ○ ○ ○ ○ ○ Know very well

**27. Do you believe that you can influence whether the herd is affected by infectious diseases or not?**

Yes, to a high degree ○ ○ ○ ○ ○ ○ ○ No, not at all

**28.** Sometimes farms are affected by outbreaks of serious infectious diseases, where the state offers economic compensation for production losses, values of animals and cleaning. By applying certain biosecurity routines, the risk to introduce some of these infections can be decreased.

**Do you think that all affected farmers should get equal compensation level, whether or not they have routines present to prevent introduction of the disease in question (e.g. through participation in a biosecurity programme)?**

| | | | | | | | | | |
|---|---|---|---|---|---|---|---|---|---|
| | | | | | | | | | Don't know |
| No, the ones without routines should not have anything at all | ○ | ○ | ○ | ○ | ○ | ○ | ○ | Yes, everyone should have the same | ○ |

**29. If you have comments to the questions above, please write here**

________________________________________
________________________________________
________________________________________
________________________________________

## C. RISK COMMUNICATION AND INFORMATION

**30. Consider the following scenario:**
*Recently, your herd has experienced diffuse problems with coughing and you decide to try and find out more about potential causes.*

**How would you prefer to access the information needed?** (Specify one or more options)

**I prefer:**

- ☐ Websites of Swedish authorities and universities
- ☐ Websites of Swedish animal health organisations and associations
- ☐ Websites of insurance companies
- ☐ Other producers web pages
- ☐ Foreign websites
- ☐ Video clips online (e.g. clips of animals with symptoms, lectures etc.)
- ☐ Discussions with friends, acquaintances, colleagues
- ☐ Industry branch journals and magazines
- ☐ Books and compendia
- ☐ Lectures and courses
- ☐ conferences and fairs
- ☐ Discussions with the farm vet
- ☐ Short summaries and pamphlets
- ☐ National phone line for medical information
- ☐ Smart phone applications
- ☐ Other

If other, please specify:

______________________________________________

______________________________________________

______________________________________________

______________________________________________

**31. Consider the following scenario:**
*A serious animal disease, never before seen in Sweden, is demonstrated at the National Veterinary Institute (SVA). In order to provide information about the outbreak and prevent further spread of infection, the authorities' disease control experts want to reach Swedish farmers as soon as possible.*

**What is the best and most effective way for them to reach you with this information? Select the options that you think are good or bad.**

| | good | bad |
|---|---|---|
| Letters by post from the authorities to animal owners | ○ | ○ |
| Updated information on the website of SVA | ○ | ○ |
| Updated information on the website of the Board of Agriculture | ○ | ○ |
| Social media (e.g. Twitter, Facebook) | ○ | ○ |
| E-mail list for spread of information from authorities to animal owners | ○ | ○ |
| Phone text messages from authorities to animal owners | ○ | ○ |
| Smartphone application | ○ | ○ |
| Local radio | ○ | ○ |
| Local information meeting | ○ | ○ |

Better reached by other means (please describe how):

______________________________

**32. If you have comments to the questions above, please write here**

______________________________
______________________________
______________________________

**33. Would you consider buying animals from herds with an unknown disease status?**
i.e. you do not know their disease morbidity level and if they are affiliated to relevant disease control programs

No, I would never do that ○ ○ ○ ○ ○ ○ ○ Yes, that does not restrict purchase of animals

**34.** *Assume that the affiliation and status of individual herds in different control programs is summarised and made freely available online.*

**I would like the health status of my herd to be freely available to all.**

Totally disagree ○ ○ ○ ○ ○ ○ ○ Totally agree

**35. To be able to find out the health status of <u>other</u> herds is**

Very valuable ○ ○ ○ ○ ○ ○ ○ Completely unnecessary

**36. If you have comments to the questions above, please write here**

______________________________
______________________________
______________________________

## D. COMPARTMENTATION, FLOW, CLEANING

**About compartmentation**
On farms with animals for food production, there are different degrees of compartmentation of the animals (i.e., grouping and separation of different groups in different compartments). Parts of poultry production, for example, strict compartmentation with separate rooms, separate protective clothing and equipment for each department and animal groups kept together throughout the production cycle. Farms that keep all their animals in one and the same stable and does not have any kind of batch rearing lacks compartmentation.

**37. To what degree is the are livestock on the farm compartilised**

no compartmentation ○ ○ ○ ○ ○ ○ ○ strict compartmentation

Different degree for different species of animals on the farm ○

**38. If the farm does not have compartmentation, what do you think is the reason that prevents this?** (Specify one of more alternatives)

- ☐ I see no profit in this
- ☐ The structural design of stable buildings
- ☐ The type of production
- ☐ It would take too much time
- ☐ Herd size is too big
- ☐ Herd size is too small
- ☐ Not enough space
- ☐ Other reason, fill in below

________________________________________________
________________________________________________

**39. Consider the following scenario:**
*You discover widespread cough in a group of animals and want to prevent spread to the rest of the herd.*

**Do you think that you could arrange a temporary solution in order to avoid direct or indirect contact between the group with coughing and other animals during a two-week period?**

Yes, it could easily be arranged ○ ○ ○ ○ ○ ○ ○ No, it’s impossible

The farm has complete compartmentations of all animals ○

**40. Are there procedures for regular cleaning of the feed table / trough / feed chute and water cups / nipples?**

○ Yes
○ No

**41. Does movement of feed, animal and manure, or the work routines of the staff, lead to feed and drinking water being accidently contaminated with manure?**

No, never ○ ○ ○ ○ ○ ○ ○ Yes, very often

**42. If the farm has compartmentation, is it your routine to empty and clean buildings between batches?**

- ○ Yes
- ○ No
- ○ No compartmentation

**43. Is it your routine to empty and clean all animal stables at least once a year?**

- ○ Yes
- ○ No

**44. If you are the owner of the farm:**

**Who are your key advisers and information channels for new construction, renovation, or reconstruction of stable buildings?**

________________________________________

________________________________________

________________________________________

________________________________________

**45. If you have built new stables, or renovated/rebuilt stables in the last 5 years:**
**Did you consider hygiene and disease control?**
e.g. sanitation facilities, hygiene barriers, that feed do not come into contact with manure, compartmentation, quarantine housing, sanitary systems in feed or feed table, sick box, entrance for visitors, etc.

| | | | | | | | | | Have not built or renovated in the last 5 years |
|---|---|---|---|---|---|---|---|---|---|
| No consideration | ○ | ○ | ○ | ○ | ○ | ○ | ○ | Much consideration | ○ |

**46. Did anything prevent you from taking this into account?**

- ○ Yes
- ○ No

If yes, describe: What prevented you?

________________________________________

________________________________________

________________________________________

**47. Have the advisers that you have engaged in construction or renovation paid careful attention and discussed disease control and hygiene issues with you?**

Not at all ○ ○ ○ ○ ○ ○ ○ To a large extent

**48. Place for comments to the questions above**

________________________________________

________________________________________

________________________________________

## E. PROTECTIVE CLOTHING FOR PROFESSIONALS VISITING ANIMAL STABLES

The following part of the questionnaire includes in depth questions dealing with stable visitors and use of protective clothing when visitors enter stables, or have contact with the animals on the farm.

The question layout follows a behavioural science method which involves many seemingly similar questions. This is needed in order to capture all influencing factors. Protective clothing is ONE example of different biosecurity measures possible. Another example is control of animal trade, but this and other issues, is something that we investigate in other studies.

By **professionals visiting farms**, we mean people who enter livestock stables or come into contact with livestock as part of their profession, but do not work or are employed at the farm. Examples of professionals visiting farms are **craftsmen, veterinarians, transporters, advisers, inseminators** or **inspectors**.

By protective clothing, we mean **boots or shoe covers**, and **coats or coveralls** that are designed to prevent spread of infections - i.e. not clothes used to protect persons working in the stables. It may be the visitor's own clean (washed or disposable) protective clothing or protective clothing offered by the farm.

**49. Do you have protective clothing for professionals visiting the farm, indicate what is available on the farm**

- ☐ Coveralls
- ☐ Coat
- ☐ Boots
- ☐ Shoe covers
- ☐ Do not have protective clothing for visitors

**IMAGINE THE FOLLOWING SITUATIONS AND RESPOND TO HOW YOU WOULD HAVE ACTED:**

**50.** *Your usual animal transporter did not have time so a transporter that you don't know will pick up animals from the farm. Apparently, the transporter has been to another farm earlier in the day and is now about to step into the stable to help with the loading, without putting on protective clothing.*

**Do you ask the transporter to put on protective clothing?**

No, not likely ○ ○ ○ ○ ○ ○ ○ Yes, very likely — Never let transporters enter the stable ○

**51.** *An acquaintance has asked you to welcome a workplace group who wants to do a field trip to a farm. You don't know the background of individual visitors or if they have animals at home. When they arrive, it turns out that they have forgotten their shoe covers and disposable coveralls that they have obtained in order to "not become dirty", and it would take them fifteen minutes to go back and fetch these*

**Would you let them enter the stables without protective clothing?**

No, not likely ○ ○ ○ ○ ○ ○ ○ Yes, very likely — Never allow field trips to my farm ○

**52.** *A repairman who travels between farms is about to enter the animal stables without changing clothes or shoes.*

**Do you let the repairman enter without putting on protective clothing?**

No, not likely ○ ○ ○ ○ ○ ○ ○ Yes, very likely

**53.** *It is a Sunday morning and you have been forced to call for a vet. It's not your ordinary vet but someone new who you have not met before and who seems stressed from other emergency calls waiting. You can see that the vet puts on a clean coat but the vet's shoes are not properly cleaned – there are traces of manure on them.*

**Do you ask the vet to change or clean the boots before the vet enters the stables?**

No, not likely ○ ○ ○ ○ ○ ○ ○ Yes, very likely

**54.** A new neighbour who has started recently started a hobby farm with animals of the same species as yours comes by your farm in her stable clothes to ask you something. You are busy inside the stable and cannot come out to the yard.

**Do you ask your neighbour to put on protective clothing before entering the animal stables?**

No, not likely ○ ○ ○ ○ ○ ○ ○ Yes, very likely

**55.** *A salesman who wants to offer a product that you are interested in is visiting your farm. The salesman looks clean but wears leather shoes that seem difficult to wash.*

**Do you ask the salesman to put on protective clothing before he enters the animal stable?**

No, not likely ○ ○ ○ ○ ○ ○ ○ Yes, very likely

I never let salesmen enter the stables ○

**56.** A neighbours' children *(who does not have production animals at home) want to come in and have look in the stables. They bring a couple of friends from school that you haven't seen before and you don't know if they have animals at home.*

**Do you ask the children to put on protective clothing before they enter the animal stables?**

No, not likely ○ ○ ○ ○ ○ ○ ○ Yes, very likely

I don't let the children enter the stables ○

**57.** You have heard that there is an ongoing outbreak with serious coughing and sickness among animals at some farms within your business branch. One of your acquaintances, who has animals of the same species as you, wants to come in for a chat and have a look at your animals.

**Do you ask your acquaintance to put on protective clothing before he enters the stables?**

No, not likely ○ ○ ○ ○ ○ ○ ○ Yes, very likely

I don't let the visitor enter the stables ○

**58. Place for comments to the questions above**

____________________________________________________________________________________________

____________________________________________________________________________________________

____________________________________________________________________________________________

____________________________________________________________________________________________

By **professionals visiting farms**, we mean e.g. **craftsmen, veterinarians, transporters, advisers, inseminators** or **inspectors**.

By protective clothing, we mean **boots or shoe covers**, and **coats or coveralls**. It can may be protective clothing offered by the farm or the visitor's own clean protective clothing.

**Note that elements of the TPB methodology was not given in the original questionnaire.**

**59. To make sure that professionals visiting the farm use protective clothing is**

| | | | | | | | | |
|---|---|---|---|---|---|---|---|---|
| Of great benefit | ○ | ○ | ○ | ○ | ○ | ○ | ○ | Not of any benefit at all |
| Cumbersome | ○ | ○ | ○ | ○ | ○ | ○ | ○ | Easy |
| Essential | ○ | ○ | ○ | ○ | ○ | ○ | ○ | Unimportant |

**Direct measures of Attitude**

**60. To make sure that professionals visiting the farm use protective clothing is unnecessary because the health status of animals in Sweden is so good**

Do not agree at all ○ ○ ○ ○ ○ ○ ○ Agree completely

**61. Professionals visiting the farm will think that I'm being difficult if I ask them to use protective clothing**

**Behavioral Belief**

Unlikely ○ ○ ○ ○ ○ ○ ○ Very likely

**62. If professionals visiting the farm use protective clothing or not will influence the health status of the herd**

**Behavioral Belief**

No, not likely ○ ○ ○ ○ ○ ○ ○ Yes, very likely

**63. To make sure that professionals visiting the farm wear protective clothing will decrease the risk that my herd will suffer from outbreaks of infectious diseases**

**Behavioral Belief**

Unlikely ○ ○ ○ ○ ○ ○ ○ Very likely

**64. Professionals that visit the farm will think that I don't trust them if I ask them to use protective clothing**

**Behavioral Belief**

Unlikely ○ ○ ○ ○ ○ ○ ○ Very likely

**65. Place for comments to the questions above**

______________________________________________________________________________

By **professionals visiting farms**, we mean e.g. **craftsmen, veterinarians, transporters, advisers, inseminators** or **inspectors**.

By protective clothing, we mean **boots or shoe covers**, <u>and</u> **coats or coveralls**. It can may be protective clothing offered by the farm or the visitor's own clean protective clothing.

**66. The animals' health is very important to me**

Outcome Evaluation

Do not agree at all ○ ○ ○ ○ ○ ○ ○ Agree completely

**67. To keep the herd free from infectious diseases is very important to me**

Do not agree at all ○ ○ ○ ○ ○ ○ ○ Agree completely

Outcome Evaluation

**68. I really don't want professionals visiting the farm to think that I'm being difficult**

Do not agree at all ○ ○ ○ ○ ○ ○ ○ Agree completely

Outcome Evaluation

**69. If professionals that visit the farm think that I'm being difficult this could have a negative effect on the service that they give**

No, not likely ○ ○ ○ ○ ○ ○ ○ Yes, very likely

Outcome Evaluation

**70. It is important to me to have a good relationship with the professionals that visit the farm and that they don't think that I distrust them**

Do not agree at all ○ ○ ○ ○ ○ ○ ○ Agree completely

Outcome Evaluation

**71. It is expected of me that I make sure that professionals that visit the farm wear protective clothing**

Direct measure of Subiective Norm

Do not agree at all ○ ○ ○ ○ ○ ○ ○ Agree completely

**72. Place for comments to the questions above**

______________________________________________________________________________
______________________________________________________________________________
______________________________________________________________________________
______________________________________________________________________________

By **professionals visiting farms**, we mean e.g. **craftsmen, veterinarians, transporters, advisers, inseminators** or **inspectors**.

By protective clothing, we mean **boots or shoe covers**, and **coats or coveralls**. It can may be protective clothing offered by the farm or the visitor's own clean protective clothing.

**73. I feel a pressure from people in my environment to make sure that professionals that visit the farm wear protective clothing**

Do not agree at all ○ ○ ○ ○ ○ ○ ○ Agree completely

Direct measure of Subiective Norm

**74. People that are important to me want me to make sure that professionals that visit the farm wear protective clothing**

Do not agree at all ○ ○ ○ ○ ○ ○ ○ Agree completely

Direct measure of Subiective Norm

**75.** If you are the owner of the animals:
**My close family and friends think that it is important that professionals that visit the farm wear protective clothing**

Normative Belief

Do not agree at all ○ ○ ○ ○ ○ ○ ○ Agree completely

I'm not the owner of the animals ○

**76. In general, how much to you care what your close family and friends think about what you do**

Not at alll ○ ○ ○ ○ ○ ○ ○ Very much

Motivation to Comply

**77. The farm vets think that professionals that visit the farm should wear protective clothing**

Do not agree at all ○ ○ ○ ○ ○ ○ ○ Agree completely

Normative Belief

**78. If the farm vets have opinions about what you do: how much do you care?**

Not at all ○ ○ ○ ○ ○ ○ ○ Very much

Motivation to Comply

**79.** If you are employed at the farm:
**My employer/head of staff think that it's important that I make sure that professionals who visit the farm wear protective clothing**

Do not agree at all ○ ○ ○ ○ ○ ○ ○ Agree completely

Not employed ○

Normative Belief

**80.** If you are employed at the farm
**In general, how much do you care what your employer/head of staff thinks about what you do**

Not at all ○ ○ ○ ○ ○ ○ ○ Very much

Not employed ○

Motivation to Comply

**81. Within my branch of farming it is considered important that professionals that visits farms use protective clothing**

Do not agree at all ○ ○ ○ ○ ○ ○ ○ Totally agree **Normative Belief**

**82. Is it important to you to follow the industry guidelines?**

Very important ○ ○ ○ ○ ○ ○ ○ Not at all important **Motivation to Comply**

**83. The farm neighbours that have production animals think that professionals that visit farms should use protective clothing**

**Normative Belief**

Do not agree at all ○ ○ ○ ○ ○ ○ ○ Totally agree

Has no neighbours with production animals ○

**84. I care about what the neighbours with production animals think about what I do**

Not at all ○ ○ ○ ○ ○ ○ ○ Very much

Has no neighbours with production animals ○

**Motivation to Comply**

**85. Swedish authorities with a link to production animals (e.g. the National Veterinary Institute, the Board of Agriculture, and the County Board) think that it is important that professionals that visit farms use protective clothing**

Do not agree at all ○ ○ ○ ○ ○ ○ ○ Totally agree **Normative Belief**

**86. What Swedish authorities with a link to production animals (e.g. the National Veterinary Institute, the Board of Agriculture, and the County Board) think about what I do is important to me**

Not at all ○ ○ ○ ○ ○ ○ ○ Very much **Motivation to Comply**

**87. Place for comments to the questions above**

______________________________________________________________________________
______________________________________________________________________________
______________________________________________________________________________
______________________________________________________________________________

By **professionals visiting farms**, we mean e.g. **craftsmen, veterinarians, transporters, advisers, inseminators** or **inspectors**.

By protective clothing, we mean **boots or shoe covers**, <u>and</u> **coats or coveralls**. It can may be protective clothing offered by the farm or the visitor's own clean protective clothing.

**88. To make professionals that visit the farm use protective clothing is entirely up to me**

Do not agree at all ○ ○ ○ ○ ○ ○ ○ Totally agree

**Direct measure of Perceived Behavioral Control**

**89. To make professionals that visit the farm use protective clothing is**

Impossible ○ ○ ○ ○ ○ ○ ○ Possible

**Direct measure of Perceived Behavioral Control**

**90. I'm convinced that I could make professionals that visit the farm use protective clothing if I wanted to**

Do not agree at all ○ ○ ○ ○ ○ ○ ○ Totally agree

**Direct measure of Perceived Behavioral Control**

**91. In general, is it your experience that you are able to influence how professionals that visit the farm behave when they are at the farm?**

Never ○ ○ ○ ○ ○ ○ ○ Always

**Direct measure of Perceived Behavioral Control**

**92. Professionals that visit the farm are often in a hurry**

Do not agree at all ○ ○ ○ ○ ○ ○ ○ Totally agree

**Control Belief**

**93. When professionals that visit the farm are in a hurry, the probability is...**

**Control Belief Power**

high ○ ○ ○ ○ ○ ○ ○ low **...that I ask them to use protective clothing**

**94. It happens frequently that professionals that visit the farm enter the animal stables without someone from the farm being present**

Do not agree at all ○ ○ ○ ○ ○ ○ ○ Totally agree

**Control Belief**

**95. By using an obvious hygiene barrier and/or instructions, I can usually make professionals that visit the farm use protective clothing, even if no one from the farm is present when they enter the stables**

Do not agree at all ○ ○ ○ ○ ○ ○ ○ Totally agree

Cannot answer ○

**Control Belief Power**

**96. Place for comments to the questions above**

______________________________

______________________________

______________________________

______________________________

By **professionals visiting farms**, we mean e.g. **craftsmen, veterinarians, transporters, advisers, inseminators** or **inspectors**.

By protective clothing, we mean **boots or shoe covers**, and **coats or coveralls**. It can may be protective clothing offered by the farm or the visitor's own clean protective clothing.

**97. Is it your experience that professionals that visit the farm don't think that they need to use protective clothing?**

Never ○ ○ ○ ○ ○ ○ ○ Always

**Control Belief**

**98. If a professional who visits the farm don't think that it's necessary to use protective clothing, the probability is...**

**Control Belief Power**

high ○ ○ ○ ○ ○ ○ ○ low **...that I can make that visitor use protective clothing**

**99. Does it happen that protective clothing offered at the farm does not fit the visitor because the size is wrong?**

That never happens ○ ○ ○ ○ ○ ○ ○ It happens very often

The farm does not offer visitors protective clothing ○

**Control Belief**

**100. It is difficult for me to make a visitor use protective clothing if the size is wrong**

Do not agree at all ○ ○ ○ ○ ○ ○ ○ Totally agree

The farm does not offer visitors protective clothing ○

**Control Belief Power**

**101. It is difficult and takes a lot of time to keep protective clothing clean**

Do not agree at all ○ ○ ○ ○ ○ ○ ○ Totally agree

**102. Protective clothing offered at the farm is often dirty**

Do not agree at all ○ ○ ○ ○ ○ ○ ○ Totally agree

The farm does not offer visitors protective clothing ○

**Control Belief**

**103. It would be difficult for me to make a visitor use protective clothing if it was dirty**

Do not agree at all ○ ○ ○ ○ ○ ○ ○ Totally agree

**Control Belief Power**

**104. It is expensive to offer protective clothing to all professionals that visit the farm**

Do not agree at all ○ ○ ○ ○ ○ ○ ○ Totally agree

**Control Belief**

**105. Do costs influence the choice to offer (or not to offer) visitors protective clothing?**

To a high degree ○ ○ ○ ○ ○ ○ ○ Not at all

**Control Belief Power**

**How did you first come into contact with this questionnaire?**

- ○ It was posted by mail
- ○ It was sent by e-mail
- ○ I found it on Internet

**If you have any other comments, please write here**

________________________________________
________________________________________
________________________________________
________________________________________
________________________________________
________________________________________
________________________________________
________________________________________

## THANK YOU for participating in this study!
